# Enhancing the clinical research workforce: a collaborative approach with human resources

**DOI:** 10.3389/fphar.2024.1295155

**Published:** 2024-02-07

**Authors:** Denise C. Snyder, Heather Gaudaur, Mark Marchant, Laura Viera, Andrea McCubbin, William Verble, Angela Mendell, Christine Gilliam

**Affiliations:** ^1^ Duke Office of Clinical Research, Duke University, Durham, NC, United States; ^2^ School of Medicine, Duke University, Durham, NC, United States; ^3^ Clinical and Translational Science Institute, Duke University, Durham, NC, United States; ^4^ Rewards and Recognition, Office of Human Resources, Duke University, Durham, NC, United States; ^5^ Clinical Trials Administrative Offic, University of Alabama at Birmingham (UAB), Birmingham, AL, United States; ^6^ North Carolina Translational and Clinical Sciences Institute, University of North Carolina at Chapel Hill (UNC-CH), Chapel Hill, NC, United States; ^7^ Office of Research, College of Medicine, University of Kentucky (UK), Lexington, KY, United States; ^8^ Human Resources, University of Kentucky (UK), Lexington, KY, United States; ^9^ Center for Clinical and Translational Science and Training (CCTST), College of Medicine, University of Cincinnati (UC), Cincinnati, OH, United States; ^10^ College of Medicine Human Resources, University of Cincinnati (UC), Cincinnati, OH, United States

**Keywords:** clinical research professional (CRP), clinical research coordinator (CRC), clinical research nurse (CRN), human resources (HR), workforce development, academic medical center (AMC)

## Abstract

Jobs for clinical research professionals (CRPs) have grown increasingly complex over the past 20+ years. This is due largely to additional administrative burden for investigators, study teams, sponsors, Clinical Research Organizations (CROs), and sites, particularly Academic Medical Centers (AMCs). Furthermore, National Institutes of Health (NIH) has reduced capacity to effectively fund research recognizing this is dependent on the overall congressional budget, which creates greater pressure for clinician scientists to secure external support. It is widely known clinical research will continue to become increasingly more complex for clinician scientists. This manuscript explores adoption of a clinical research competency-based job classification framework from the Joint Task Force for Clinical Trial Competency (JTFCTC) across several AMCs and the role of Human Resources (HR) in facilitating this process. This collaboration focuses on fostering successful projects tied to the business case in order to address equity and improve support for the clinical research enterprise.

## 1 Introduction

In today’s rapidly evolving landscape, clinical research sites, particularly those in AMCs, need to revitalize job descriptions and establish career pathways (Brouwer et al., 2017). Such efforts aim to reduce turnover, increase employee engagement, and improve clinical trial quality (Stroo et al., 2020). Staff supporting the research for clinician scientists are asked to do more than recruit participants and complete study visits, while also facing an increased regulatory burden and a fragmented infrastructure (Sung et al., 2003). Although job responsibilities have evolved, the job titles, essential skills, and salaries held by individuals performing these tasks have not (Knapke et al., 2022). In addition, training demands have soared and resources for professional development remain limited. Associated costs with training are already substantial (Deeter et al., 2020). However, with ill-defined jobs, it is often arduous to garner how many clinical research professionals (CRPs) are hired (Bocchino et al., 2020), thus actual costs of training have likely been underestimated. These CRP jobs are in high demand, with increasingly documented shortages related to a workforce in crisis (Freel et al., 2023).

Beginning in 2014, the Association for Clinical and Translational Science (ACTS) Clinical Research Professional Taskforce issued recommendations advising AMCs to assess the training, support, and career development needs of CRPs (Speicher et al., 2012; Sonstein et al., 2014; Stevens and Daemen, 2015; Knapke et al., 2022). Competency-based job frameworks provide a foundation to integrate and enhance recruitment, development, performance management, and career progression (Benayoune, A. 2017). To affect change, a deep dive is needed by AMCs with representation by stakeholder groups requiring engagement across the institution for departments, faculty, staff and administrators (Snyder et al., 2016). Critical partnerships and relationships must be established between institutional clinical research leadership and leaders in Human Resources (HR) (Brouwer et al., 2017). Successful competency-based job models for clinical research professionals have been demonstrated by several institutions (Furtado et al., 2015; Brouwer et al., 2017; Deeter et al., 2020; Ibrahim et al., 2022). In this paper, we will describe elements of successful institution-wide partnerships focusing specifically on the importance of engaging HR in both private and public AMCs. We will provide key recommendations spanning various phases of competency-based job framework adoptions highlighting areas of success, challenges, and lessons learned.

## 2 How to get started

Revamping job descriptions and creating career ladders in any industry is daunting. Doing it in a traditional AMC clinical research setting where teams have operated in a decentralized, siloed manner may seem impossible. Despite the challenges, endeavors to standardize the CRP positions and career pathways exist using the Joint Task Force for Clinical Trial Competency framework https://mrctcenter.org/clinical-trial-competency/ (Sonstein et al., 2014; Kolb et al., 2018; Sonstein et al., 2018; Musshafen et al, 2021) and are well-documented by Duke University (Brouwer et al., 2017; Deeter et al., 2020; Stroo et al., 2020), and further instantiated at the University of Alabama at Birmingham (UAB) in March 2020. More recently, various efforts are underway at other AMCs, several included in this perspective. There are likely other implementations by AMCs in-part, or in-whole that are not known or documented.

To initiate the competency-based job classification project, organizations must make an effective business case to institutional leadership, clearly articulating the benefits of revising job descriptions and career pathways to attract, retain and motivate staff. This involves demonstrating how a project of this caliber directly aligns with organizational goals including increased research funding, high quality staff support, long-term reduced administrative costs and improved competitiveness in the clinical research industry, spanning both jobs and funding. This step may vary slightly across institutions, however, to fully understand the institutional landscape, an assessment of the existing clinical research workforce is needed. This assessment may include the following: 1) Define the CRP role and identify existing jobs supporting clinical research; 2) Review current job descriptions and corresponding salary ranges; 3) Solicit feedback from CRPs, managers, and clinician scientists on the current tasks and competencies needed to perform CRP jobs; 4) Identify deficits in current workforce management process; and 5) Review literature and attend sessions on existing competency-based clinical research job frameworks. This data-driven approach sets the stage for success by providing the foundation for future conversations with key stakeholders.

**FIGURE 1:**
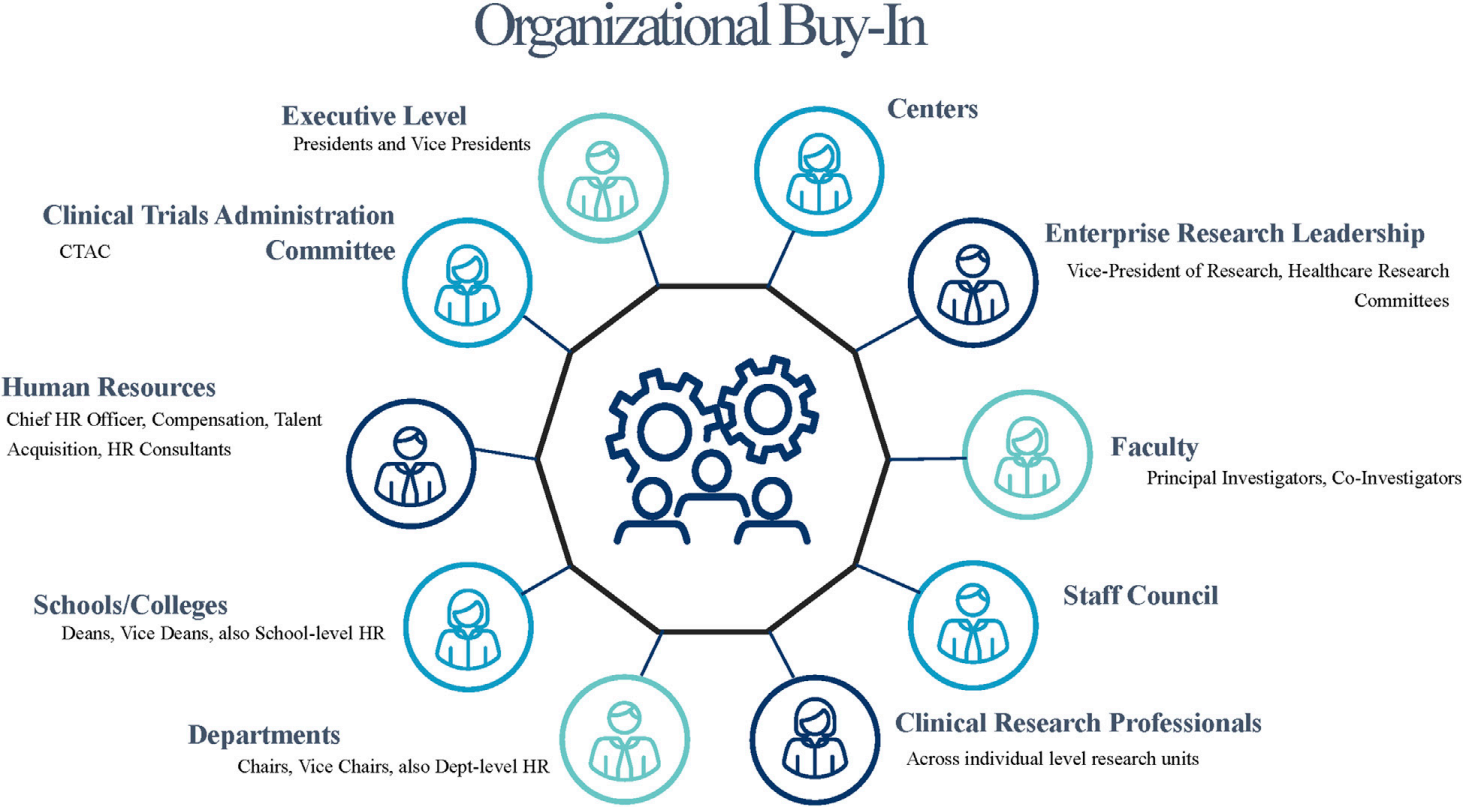
Organizational buy-in is needed across key institutional stakeholders.

The next step includes buy-in and partnership from key stakeholders, including (but not limited to, given differences across organizations): Clinical research leadership, School and departmental leadership, HR (including Compensation and Recruitment), Faculty, and Staff (See Figure 1). At Duke, in the second and successful attempt at job classification revision and implementation, buy-in and partnership was sought from top leadership. This began with Vice Deans for Clinical Research, Finance, and HR before proceeding to leadership in each clinical research unit (24 units align with clinical areas). UAB’s approach mirrored Duke’s by initiating conversations initially with senior leadership within the Heersink School of Medicine, where the majority of the affected staff’s positions resided, before making the pitch to the Chief HR Officer for the institution. Once approval was garnered at those levels, the campaign to disseminate high level information about the upcoming effort commenced with the University’s institutional-wide Clinical Trials Administration Committee and then diffused from there. Similarly, at University of North Carolina at Chapel Hill (UNC-CH), a survey of School of Medicine (SOM) CRPs revealed that the area of lowest job satisfaction among respondents related to lack of clear career pathways and highlighted the importance of such an initiative to SOM and HR leaders. At the University of Kentucky (UK), leadership buy-in was initially achieved by the College of Medicine (COM) Office of Research establishing a Research Professionals Network encompassing CRPs in COM and the Center for Clinical and Translational Sciences (CCTS). CRPs were invited to share challenges and barriers to carry out daily tasks responsibly and effectively. Feedback included non-competitive salary ranges, misaligned job responsibilities, lack of training opportunities, and lack of a defined career pathway. Using this feedback as the catalyst for redefining the CRP job architecture demonstrated COM’s commitment and alignment with the institution’s strategic plan principle to “Take Care of our People” (https://pres.uky.edu/strategic-plan). At the University of Cincinnati (UC), there were narrowly focused efforts to address compensation while battling increased turnover with little focus on other factors contributing to retention. For example, clinical research leaders were aware of shortcomings in the CRP job classification framework, but little work had been done to evaluate competency-based job models and career advancement. UC took a team science approach to addressing these issues. Team science brings together people from different fields and utilizes their expertise in a collaborative manner to tackle projects or issues (NIH, National Cancer Institute Division of Cancer Control and Population Sciences, 2021). UC’s approach was to form a CRP workgroup in which membership had a cross-section of contributors from UC’s CCTST, College Human Resources, CRP leaders, and faculty. This group was charged by its Sr. Associate Dean of Clinical Research. UC’s approach is 3-pronged exploring Education, Recruitment and Retention. Each group is focusing on specific initiatives within an area and is functioning independently, yet collaboratively, to improve each of these areas. A major endeavor of the CRP workgroup is to incorporate competency-based job descriptions and create career advancement pathways for CRPs.

Identifying the “win-win” reasons for establishing a competency-based framework was a key to success in prior implementations. Duke reduced staff turnover by 30% (Stroo et al., 2020) and improved professional development and career advancement (Deeter et al., 2020). This structure allows the organization to capitalize on the data associated with these jobs, while attracting more diverse faculty expertise and increasing the institution’s clinical research portfolio. Prior to UAB’s implementation in 2020, it was not able to identify, much less track, its CRP workforce given the more than 80 job titles used across the institution. Now, the University is able to monitor its growth in the workforce, which has shown a 20% increase over the past year. Likewise, UAB is now able to monitor its retention rate of CRPs and ensure communications and training opportunities reach their target audience.

## 3 The importance of a human resources (HR) collaboration

An overhaul of clinical research job classifications requires a vital HR partnership. HR professionals play a crucial role in talent management and workforce planning and engaging them early and often throughout the process facilitates a smooth implementation. Establishing a strong rapport involves educating HR about the unique job requirements of the clinical research field, cost of turnover, including lengthy time to fill positions and subsequent training, and workload demands for managers. In addition, this partnership ensures HR professionals understand the institutional infrastructure for oversight and support, fosters open communication channels, and helps them understand the similarities and differences for clinical research staff compared to jobs in private industry and patient care. In turn, the CRP workgroups learn about HR-related themes such as market analysis for compensation, salary transparency, equity across and within institutional organizations, as well as practices that may relate to recruiting and compensating staff outside the landscape. The workgroup collaborates on understanding the organization’s processes. Each institution has its own policies and processes related to job design and compensation practices. Working in partnership with HR ensures compliance, efficiency, and equity to meet project timelines. Engaging HR early during the needs assessment phase allows the HR team to identify potential barriers to proactively address.

Building on the assessment of the institutional landscape as the foundation to engage key stakeholders, HR partners can assist greatly. This step includes identifying clinical research facilities or departments across the various areas within the project’s scope and determining how many staff are in those areas along with respective titles. This can be achieved by reviewing multiple sources of HR data (University, School, and/or Department). Duke started with a list of employees named as key personnel in protocols submitted to its University Health System’s Institutional Review Board (IRB), and then reviewed employees in frequently used positions. For UAB, this meant reaching out to the HR and administrative officers in each School to confirm or deny that clinical research was being conducted there and provide the staff names along with corresponding title, organization, and supervisor. At UNC-CH, early data was obtained using a custom-developed *Profile and Training System* (*PaTS*) in which all employees engaged in clinical research were asked to indicate their primary role. This identified significant variance in roles compared to job classifications, further emphasizing the need for standardization. A combination of self-reported data from PaTS, job classification data from HR, study role information from IRB submissions, and supervisor-reported data will be used for the final, comprehensive identification of CRPs. UK accomplished this by defining a “clinical research professional” and then identifying job titles likely to have associated responsibilities. HR consulted with the project advisory group, comprised of COM leadership, CRPs, CCTS, and Cancer Center representatives, to identify which job titles and employees were in scope for the project. UC’s existing structure had all CRPs identified, but the job descriptions and career framework were lacking. UC CRP leaders suspected missing job classifications (i.e., Nurse Research, Regulatory) and HR assisted with reviewing these prior to the career pathway work.

## 4 Revision of jobs and adopting the JTFCTC framework

Using a data-driven assessment, the workgroup evaluates the current job descriptions and information gathered to identify gaps and areas for improvement. This will vary across institutions. At Duke, this was enterprise-wide for Schools of Medicine and Nursing that support biomedical research (Brouwer, et al., 2017). UAB’s effort took an institutional-wide perspective by including all seven Schools and Colleges engaged in the conduction of clinical research. In addition to Medicine, this encompassed Dentistry, Optometry, Nursing, Public Health, Health Professions, and Arts & Sciences. At UNC-CH, a public state institution, it was critical to develop the standardized position descriptions in a manner that would align with the existing state of North Carolina career banding profiles. To ensure the standardized positions would be acceptable based on those statewide requirements and standards, individuals from the UNC-CH SOM first worked closely with HR representatives to develop the position descriptions and then reviewed the proposed positions with leaders, managers, and staff from across the SOM to fine-tune the descriptions. At UK, the advisory group formed a workstream specific to each job title [CRC, Clinical Research Nurse (CRN), Regulatory Specialist, etc.] comprised of CRPs in that role to conduct the job description reviews. This ensured a larger span of input and engagement in the review without having too large of an advisory group. HR representatives then provided feedback and guidance on draft job descriptions and compensation impact and considerations. In 2017, UC had identified all clinical research staff with department business leaders and managers where employees were mapped to a general CRP title and job description. UC HR and the CRP Taskforce are incorporating the framework by mapping these competencies to the existing and newly expanded job titles. UC opted to follow UNC-CH’s lead and work within the existing framework to create competency-based job descriptions and establish advancement pathways. The retention subgroup at UC is incorporating competencies into job descriptions and tiers using Duke’s tier advancement model and integrating with the advancement pathway.

As part of this work, consideration should be given for establishing career ladders, as this has been linked to retention (Stroo et al., 2020). HR can provide guidance on existing job ladders at your institution by aligning the number of levels to other ladders. Understanding the existing HR framework is critical at public institutions where there may be less flexibility due to statewide standards. Mapping career progression opportunities and defining career growth stages using the JTFCTC framework to establish job ladders is a significant step in identifying a clear career pathway for CRPs. Linking the revised job descriptions to the identified career pathways through incorporating competency skills trainings and requirements at each stage will ensure alignment for growth opportunities.

In summary, revising CRP job architecture and adopting the JTFCTC framework requires workgroups to do the following: 1) Update job classifications and titles, working with HR partners to develop associated external market-based and internally aligned salary ranges to accompany job classifications; 2) Map existing positions to revamped job classifications, carefully review the function of the role (not the individual currently in the role) to determine classification; 3) Review financial impact of potential salary adjustments with department business managers, 4) Craft letters that will notify each employee of new position title and associated compensation, 5) Retire old job descriptions, post new job descriptions; 6) Provide resources for questions and assistance (FAQs, Tip sheets, Central email/voicemail box, Town Halls, etc.); and 7) Establish data acquisition plan for tracking the revised jobs framework to measure project successes and to pivot in real-time, if needed (may involve working with data analytics team, recruitment and/or payroll services). All the institutions participating in this manuscript have learned from each other and the extensive resources and tools provided by Duke as part of their Workforce Engagement and Resilience program (https://medschool.duke.edu/research/research-support/research-support-offices/duke-office-clinical-research-docr/workforce-3).

## 5 Implementation and evaluating changes

During the planning and implementation of any project, communication is paramount. This, in fact, cannot be overstated. Stakeholders must be informed about the data-driven process resulting in revised job classifications and established career pathways to ensure understanding for individual impact, overall project transparency, and the investment of the institution. Providing necessary training to HR professionals and clinical research managers is important to implementation of the new framework. Both managers and HR professionals can assist the project team in fielding questions and triaging any problems that arise. Along with consistent and transparent communication, change management and dealing with expectations for study teams is critical for success. Not all institutions can make all the changes at once. Incremental changes along the way can assist in the longer-term plan to implement career ladders and clearer pathways for advancement. Early, easy wins can provide momentum to tackle the larger goal. For example, tackling clearer roles like nursing and regulatory tracks may provide short-term deliverables and keep institutional buy-in strong. After implementation, regularly assessing the effectiveness of the changes allows for continuous improvements and refinements to the process. A great deal of work goes into aligning clinical research job responsibilities with a competency-based framework; it will not happen overnight, and it will not be perfect for all stakeholders. The idea of perfection can be paralyzing, so best to adhere closely to the timeline, launch, seek and apply feedback, adjust the process, and make improvements. Demonstrating progress in this process communicates value to CRPs.

## 6 Conclusion

**FIGURE 2:**
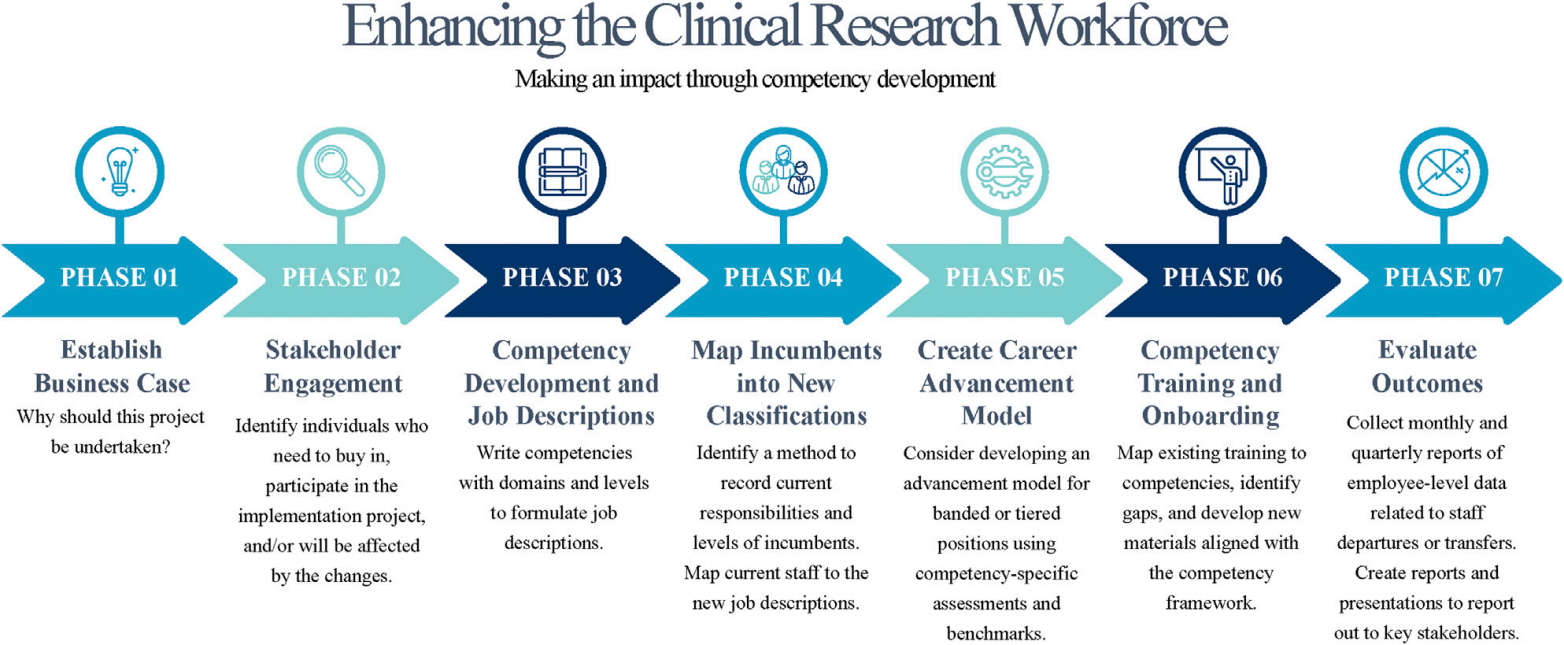
High-level steps to implementing competency-based clinical research professional jobs.

Collaboration between clinical research leadership and HR is critical for establishing and maintaining a strong workforce, and for successfully implementing competency-based job descriptions and creating career pathways. The process does not have to be overwhelming and can be mitigated by leaning on institutions with experience in this space. This paper demonstrates collaborations of five institutions working together to learn from one another and build a stronger research workforce by leveraging partnerships. By investing in this process, organizations can recognize and foster a high-quality clinical research workforce, boost employee engagement, and secure support for the growing number of clinical trials. Figure 2 provides a high-level overview on moving this process forward. Reworking the competency-based job classifications provide an excellent starting point to tie together improved onboarding, training, on the job support, expansion of diversity and inclusion efforts (Cranfill et al., 2022) and professional development (Deeter et al., 2020). Establishing strong collaborations between clinical research leadership and HR will promote building a talented workforce supporting the quality and success of clinical trials.

## Data Availability

No data sets were used in this article, but we do provide access to our tools such as a REDCap data dictionary that contains Code or technology also methodology. This data can be found here. https://medschool.duke.edu/research/research-support/research-support-offices/duke-office-clinical-research-docr/workforce-3.
