# Supplementary material for: Enhancing the clinical research workforce: a collaborative approach with human resources
Source: Front Pharmacol. 2024 Feb 7;15:1295155. doi: 10.3389/fphar.2024.1295155 (PMC10879940; doi:10.3389/fphar.2024.1295155)
Supplement: Supplementary file 1 [file DataSheet1.docx]

Okay

**Competency Framework Implementation Checklist**

Click or tap here to enter text.**’s Onboarding Learning Plan**

**This document outlines the steps taken by the Duke Office of Clinical Research within the Duke School of Medicine to implement a competency framework for the clinical research professional workforce at the university.**

| Phase 1: Establish Business Case | | |  |
| --- | --- | --- | --- |
|  | *Provide a business case for the Institution to take on the project* | Examples for Consideration:   - Review literature - Describe the potential investment - Name key objectives - Detail the business needs - Define the project plan - Demonstrate alignment with the organization’s strategic plan | |

| Phase 2: Stakeholder Engagement | | |  |
| --- | --- | --- | --- |
|  | *Identify individuals who should be part of the implementation of the project* | These individuals may include:   - Human Resources (HR) - Compensation - Finance - Business Managers - Executive leaders in Business administration - Clinical Research Finance - Faculty - Key operational leaders of central and departmental administration - Managers of clinical research professionals - Subject matter experts - Clinical research professionals | |
|  | *Identify the population* | Look for staff members with:   - Key job titles - Role-based access (e.g., key personnel in IRB system, access to Electronic Health Record) | |
|  | *Transparency and communication* | Examples may include:   - Town Halls - Web presence - Centralized email box for assistance - Formal memos from leaders and regular newsletter communications - Focus groups | |

| Phase 3: Competency Development and Job Descriptions | | |  |
| --- | --- | --- | --- |
|  | *Build framework on Joint Task Force for Clinical Trial Competency* | Domains, competencies, levels:   - Develop site-specific domains - [Insert your site]-ify competencies - Create job-specific levels (considering banding or tiering jobs to prevent internal turbulence) | |
|  | *Write job descriptions based on established competencies and levels listed above* | | |
|  | *Job descriptions with specific levels and competencies can be used to determine the position of all new hires for the institution* | Examples for consideration:   - Consider using Duke’s Title Picker tool as the starting point - Implement tool before mapping current staff. This allows for delimiting older positions. | |
|  | *Work with HR and Compensation to create levels, set salaries, and minimum qualifications for each of the job descriptions* | | |

| Phase 4: Map Incumbents into New Classifications | | |  |
| --- | --- | --- | --- |
|  | *Identify a method to record current responsibilities and levels of the population identified above* | Examples for consideration:   - REDCap tool was sent to employees for completion first, then the responses were reviewed by the manager to verify the responses provided reflected the tasks currently performed to be mapped to new position. | |
|  | *Map current clinical research staff identified to the new job descriptions using information from their manager-reviewed responsibilities list, CV, and other job documentation* | Consideration:   - The individuals assisting in moving incumbents into new job classifications should include those who are familiar with the day-to-day activities performed in clinical research. | |
|  | *Review preliminary results with leadership from each group (Department HR representative, Department clinical research leader, Department Business Manager) and adjust results as needed* | | |
|  | *Create a position effective date and implement across the enterprise* | | |

| Phase 5: Create Career Advancement Model | | |  |
| --- | --- | --- | --- |
|  | *Consider developing an advancement model for banded or tiered positions using competency specific assessments and benchmarks* | Questions to consider:   - What would signify a change in a role? - How will assessments be administered and developed? - Is there need for a more subjective leadership assessment? - The heterogeneous nature of the positions in clinical research. - Foundational competency needs and requirements if necessary. - What is feasible to operationally run and fund with the staff available? - Reoccurrence? | |
|  | *Map current clinical research staff identified to the new job descriptions using information from their manager reviewed responsibilities list, CV, and other job documentation* | Consideration:   - The individuals assisting in moving incumbents into new job classifications should include those who are familiar with the day-to-day activities performed in clinical research. | |
|  | *Disseminate information about the advancement model and process for staff and managers* | | |
|  | *Make available all assessments, learning objectives, and tools* | Tools to consider:   - A tool to determine if someone is ready to proceed with advancement - Training on how the process works - Timeline that illustrates what will occur when | |
|  | *Run a session of advancement* | Metrics and competency assignments:   - Track what competencies and levels staff are applying for - Administer any centralized assessments | |
|  | *QA the assessments submitted and disseminate results of the advancement cycle* | | |
|  | *Gather feedback on the process through surveys and focus groups* | | |
|  | *Edit process as deemed necessary* | | |

| Phase 6: Training and Onboarding | |  |
| --- | --- | --- |
|  | *Map existing training to competency framework* | |
|  | *Identify gaps and develop new training to address any gaps* | |
|  | *Develop onboarding materials for each role that are aligned with the competency framework* | |

| Phase 7: Evaluate Outcomes | |  |
| --- | --- | --- |
|  | *Extract monthly and quarterly reports of employee-level data related to staff departures or transfers to create a master file.* | |
|  | *Create reports and presentations to report out to key stakeholders.* | |
